# Supercritical Fluid Extraction of Carotenoids from Vegetable Waste Matrices

**DOI:** 10.3390/molecules24030466

**Published:** 2019-01-28

**Authors:** Micael de Andrade Lima, Ioannis Kestekoglou, Dimitris Charalampopoulos, Afroditi Chatzifragkou

**Affiliations:** Department of Food and Nutritional Sciences, School of Chemistry, Food and Pharmacy, University of Reading, Whiteknights, Reading RG6 6AH, UK; micaelmag@gmail.com (M.d.A.L.); i.kestekoglou@student.reading.ac.uk (I.K.); d.charalampopoulos@reading.ac.uk (D.C.)

**Keywords:** carotenoid extraction, supercritical CO_2_, vegetable wastes

## Abstract

The aim of this work was to evaluate a previously-developed model on supercritical fluid extraction (SFE) for carotenoid recovery from carrot peels on various carotenoid-rich fruit and vegetable wastes. To this end, 15 matrices, including flesh and peels of sweet potato, tomato, apricot, pumpkin and peach, as well as flesh and wastes of green, yellow and red peppers, were submitted to SFE under optimised conditions (59 °C, 350 bar, 15 g/min CO_2_, 15.5% (*v/v*) ethanol as co-solvent, 30 min of extraction time). The obtained extracts were characterised for their total carotenoid content, antioxidant activity and total carotenoid recovery (TCR). TCR values were greater than 90% *w/w* for most samples, with β-carotene being the most successfully extracted compound (TCRs 88–100% *w/w*). More polar carotenoids, such as lutein and lycopene, exhibited lower TCRs. A comparison with literature data suggested that carotenoid extraction is partially dependent on the composition of vegetable matrices, specifically on polysaccharide and moisture content. The results indicated that the optimised SFE conditions can be used as a general model for carotenoid extraction from various fruit and vegetable matrices and as a viable method for adding value to these waste streams by generating carotenoid-rich extracts.

## 1. Introduction

Carotenoids are molecules especially ubiquitous in red- and orange-coloured fruits and vegetables. They are a central component of human nutrition due to the important biological functions which they are involved in and responsible for [1,2,3]. These molecules are also vastly used as food colourants, have potent antioxidant activities, and can be employed as precursors of aroma or flavour compounds [4]. Due to these reasons, there is a clear interest by the food, chemical, pharmaceutical, cosmetics, personal care and nutraceutical sectors in utilising carotenoids for various applications, as functional and/or bioactive compounds.

The demand for carotenoids has been constantly increasing and the global carotenoid market is projected to register an annual growth rate of 4% from 2018 to 2023 and to surpass the USD 2000-million figure by 2023. Europe is the main market, accounting for 42% of the world total, followed by North America and Asia, which represents 25% and 20% of the figure, respectively [5]. Although most of the current commercial carotenoids are produced via chemical synthesis [4], having encompassed 76% of the market in 2014 [6], there is a significant trend towards extracting these compounds from natural sources, such as fruits and vegetables [7] and also from biomass derived from microbial fermentation processes [8]. In 2014, 24% of the global carotenoid production derived from such sources [6].

Due to the numerous disadvantages inherent in extracting carotenoids by conventional organic solvents, new methods for extracting these phytochemicals have been investigated, among which is supercritical fluid extraction (SFE). The technique has been successfully used to extract carotenoids from a range of vegetable matrices, such as pumpkin, carrot, tomato and watermelon [9,10,11,12,13,14], and also from vegetable and fruit waste matrices including banana, grape and tomato peels [15,16,17], grape, pomegranate and pumpkin seeds [18,19,20], as well as apricot bagasse and pomace [21,22].

The main aim of the aforementioned studies was to optimise the process conditions in order to achieve maximum carotenoid recovery. This is normally approached by employing either statistical designs and methodologies (e.g., Central Composite designs, Rotable designs, One-Way ANOVA) or by non-statistical methods (e.g., sequential optimisation). The key parameters investigated included the type and concentration of the co-solvent used (e.g., ethanol, methanol, hexane, acetone, isopropanol, as well as sunflower, hazelnut and canola oils), temperature, pressure, CO_2_ flow rate, sample particle size and sample moisture content. The results reported across this literature can be said not to have been presented uniformly and hence, are somewhat difficult to compare. While a few authors indicate the amount of total carotenoid recovery (TCR, % *w/w*) in the extracts in relation to the initial sample load, others limit themselves to presenting the carotenoid concentration in the extracts or their antioxidant activity, with no conclusions drawn with regards to the effectiveness of the applied method in terms of carotenoid recovery, or its efficiency compared to conventional solvent extraction methodologies, for example. As a result, direct comparisons between different matrices, techniques and models become challenging.

In a previous study [23], the extraction of carotenoids from carrot peels was optimised by Response Surface Methodology, using a Non-Factorial 2^3^ Central Composite Design of Experiments. Kinetic experiments carried out at lab scale, which were appropriately validated and modelled, enabled the optimisation of the extraction time and subsequently a study assessing the scalability potential of the method was conducted using a 10-fold higher amount of sample. Supercritical CO_2_ (S-CO_2_) derived extracts were characterised in terms of their composition and correlations were established between the conditions of extraction and the composition of the final extracts. The optimum settings for carotenoid extraction were identified as: 59 °C, 350 bar, 15 g/min CO_2_, with 15.5% (*v/v*) ethanol as co-solvent and a total extraction time of 30 min. Under these conditions, the TCR was of 87.0% (against the 86.1% figure predicted by the model) and, in the larger-scale experiments, this value reached a 96.2% mark which, to the best of the authors’ knowledge, was the highest ever reported in the literature for vegetable wastes.

Therefore, the aim of this work was to assess whether this previously-developed model for carrot peels can be applied to other fruit and vegetable matrices, with a view to employ it as a general descriptive/predictive model for the extraction of carotenoids from fruit and vegetable matrices by SFE. To this end, 15 carotenoid-rich samples, including the flesh and peels of sweet potato, tomato, apricot, pumpkin and peach, and the flesh and wastes of green, yellow and red peppers, were submitted to SFE under the optimised conditions and the obtained extracts were characterised for their total carotenoid content and antioxidant activity, whereas calculations of the TCR were also performed.

## 2. Results and Discussion

### 2.1. Sample Characterisation

The moisture and carotenoid profiling of each vegetable sample based on the solvent extraction method, as well as their total carotenoid concentration (TCC) (calculated as the sum of β-carotene, α-carotene, lutein and lycopene concentration) are presented in Table 1. In addition to the experimental data, literature data presenting the typical chemical composition of the vegetable samples in terms of total carbohydrate, protein and lipid content were collated. It should be noted that vegetable compositional data are generally scarce and, for some matrices, not available at all. Both the experimental and the literature-based figures are presented on dry weight basis to enable direct comparisons.

The most abundant carotenoid in the samples was β-carotene, which was found to be absent only in the flesh of red peppers. An earlier work reported the presence of β-carotene in red peppers [24], but at a relatively low concentration of 5.4 μg/g on a fresh weight basis (corresponding to 49.1 μg/g on a dry basis); the reason for this disparity is not clear but could be due to differences in pepper variety and/or environmental factors during cultivation and season of harvesting. On the other hand, α-carotene was only detected in apricot peels. There are reports of α-carotene in sweet potatoes [25] and in pumpkins [26], but in our samples, these only appear as traces. It is important to highlight that the carotenoid content in fruits and vegetables is highly variable even within the same variety as it is dependent on various external factors, such as type of soil, season of harvest, sun exposure, as well as state of ripening [4]. The latter can influence the carotenoid profiling [27], since some of these molecules are only formed in the last stages of ripening. Another work reported the presence of β-carotene, lutein and β-cryptoxanthin in the flesh of peach [28], however in the current study only β-carotene was detected and at very low concentrations. This signifies the importance of ripening, as in our case, this specific fruit was visibly not fully ripened at the time of analysis.

Lutein was the second most abundant carotenoid in the samples, and was absent only in pumpkin and apricot samples (flesh and peels), and in the flesh of peach. Its concentrations were much lower than those of β-carotene, except in the case of peppers, where lutein was the major carotenoid among the four analysed. In the tomato samples, lycopene was present at high concentrations, and in the case of tomato flesh, it was the most abundant carotenoid. These observations are similar to other published works [29,30].

Apart from the aforementioned discrepancies, all carotenoids analysed in this work are also reported in the literature to be present in the same samples tested, at similar concentrations [31,32,33,34].

In terms of macronutrients (carbohydrate, protein and lipid) contents in the fruit and vegetables, there can be considerable differences among matrices. However, carbohydrates overall represent the main macronutrients in all the vegetables matrices tested in this study, ranging from 55% in peppers up to 93% in tomato peels. These carbohydrates include free monomeric sugars (e.g., glucose, fructose, sucrose), fibres (e.g., cellulose, hemi-cellulose, pectin) and polysaccharides (e.g., starch). The fibre content (both the type and the concentration) is critical, as it is a key component of the fruit and vegetable cell wall and due to its complex and rigid structure, it can often make the extraction of targeted molecules by SFE challenging by limiting their dissolution into the solvent phase. The literature data show that the proteins and lipid contents also vary considerably, with the former ranging from 1.8% in tomato peels to 29.1% in pepper wastes and the latter from 1.62% in pumpkin flesh to 14.5% in tomato flesh.

### 2.2. Carotenoid Extraction by SFE

Table 2 presents the recoveries of individual and total carotenoids from the fruit and vegetable matrices on a dry weight basis by SFE using S-CO_2_ at 15 g/min and the following previously-optimised processing conditions: 59 °C, 350 bar and 15.5% (*v/v*) of ethanol for 30 min. The data presented in Table 1 were used to obtain the initial carotenoid concentration of the individual carotenoids and the total carotenoid concentration (TCC) in each sample. The total carotenoid recovery (TCR) (dry weight of TCC in the extracts/dry weight of TCC in the original sample), was calculated based on the α-carotene, β-carotene, lycopene and lutein concentrations.

Observing Table 2, it is readily noticeable that the processing conditions used for SFE are highly optimised for the extraction for α-carotene and β-carotene. In the vast majority of the matrices tested, the recovery values for both these molecules were higher than 95%. In the case of β-carotene, which was the most abundant carotenoid in the matrices, the lowest recovery values were obtained for pumpkin flesh and pumpkin peels (92.4% and 88.2%, respectively), which could be probably attributed to their more complex structures. The very high carbohydrate content of pumpkin flesh and peels (>86%, Table 1) indicate the presence of high levels of cellulose, hemicellulose and potentially pectin in its matrix, which might have hindered the diffusion of the carotenoid molecules into the CO_2_ fluid phase. Taking this into account, in cases where the fruit and vegetable tissues are rich in complex polysaccharides, the SFE process could potentially benefit from an extended extraction time, to ensure the dissolution of the remaining carotenoid molecules trapped in sites which are not easily accessible by the solvent; this happens in the later stages of the extraction and is primarily governed by diffusive mass transfer phenomena [42]. Another alternative would be to employ a slightly higher solvent flow rate, which would facilitate the extraction of these compounds.

To test this theory further, analyses of lignin were carried out in key samples (Table 3), which would enable a more complete overview of the extraction behaviour. Lignin, which fills up the spaces between cellulose, hemicellulose and pectin in the plant, is also responsible for the rigidity of the vegetable cell wall and therefore constitutes a strong physical barrier to CO_2_ diffusion in the matrix. For the sake of convenience, the results regarding TCR and moisture are repeated in Table 3. The lignin results seem to corroborate the trend observed with the results in the literature: the vegetable structure complexity, in addition to individual carotenoid polarity, also play a decisive role on the TCR values. These do not appear to be affected considerably in vegetables with less than 10% to 11% lignin. As this amount increases, notable decreases in the TCRs start to be observed.

The moisture content varies substantially among samples and claims regarding its effect are more difficult to be made. Despite the apparent stronger influence of the vegetable structure over the moisture content, the latter can sometimes be strong enough to alter the extraction behaviour. For example, the sample of tomato peels was the only matrix with a carbohydrate content higher than those of pumpkins, but in this case, the recovery of β-carotene (>96%) was higher in the former than in the latter. Similarly, lycopene, which is a major carotenoid in tomato flesh and peels (49.3% and 32.5% of TCC, respectively) and in pumpkin flesh and peels (65.3% and 47.7%, respectively), was also recovered more efficiently in the case of the tomato matrices (~95% vs. ~85%). These differences could be due to the slightly higher moisture content of tomato flesh and peels (8.1% and 6.2%, respectively) than that of pumpkin (5.9% and 5.3%), which in some previous works, was shown to result in increased TCC recoveries [21,22]. However, in order to understand to which extent the moisture content of the samples could have influenced the recovery results, further work including a detailed compositional analysis of these matrices as well as studies on their morphology are needed.

Lutein is a carotenoid that belongs to the xanthophyll group and their main difference compared to α-carotene and β-carotene is the presence of oxygen atoms, either as hydroxyl groups and/or epoxides attached to the rings in the molecule terminations, which makes them considerably more polar. This can explain the noticeably lower recovery values in the extracts compared to α-carotene and β-carotene for most matrices. In addition, it is known that xanthophylls in general can interact and bind to proteins forming heavy complexes [43], which can hinder their extraction by the supercritical fluid and consequently, lower the recoveries. In our previous optimisation work [23], we observed that a small amount of proteins was recovered from the raw samples in the extracts (~30%). An exception to this trend was the pepper samples, where lutein was almost completely recovered. This might be due to the comparatively low carbohydrate content of peppers (Table 1), reflecting a less rigid cell wall structure, which could have resulted in higher mass transfer rates and consequently, higher recoveries.

The sample mix was prepared by mixing an equal amount of each of the 14 vegetable samples and was tested in order to simulate a real industrial scenario or a conceptual SFE process for the processing of fruit and vegetable waste. The rationale behind this experiment lays on the fact that it is likely that an SFE waste extraction plant could be applicable and economically feasible within an industrial establishment/processor that aims to recover and exploit value-added components from multiple vegetable- and fruit-derived by-products and waste streams. The aim was to evaluate the potential of the established SFE extraction protocol to deliver high yields of carotenoids from a mixed source, instead of a specific vegetable matrix. All previously identified carotenoids were recovered at considerably high levels (74% to 99%) (Table 2). This indicates that the efficiency of the process was not affected by the simultaneous extraction of mixed fruit and vegetable matrices. Taking this into account and also the fact that the total carotenoid recovery and particularly that of β-carotene (the most ubiquitous carotenoid in the vast majority of the matrices) was, for all matrices, above 85% and in many, above 95% TCR (including the sample mix), a strong argument can be made for establishing SFE as a comparable unit operation for the industry. These findings demonstrated that SFE is a viable alternative to conventional extraction techniques, and potentially a viable method for adding value to vegetable waste streams by generating carotenoid-rich extracts from these.

The antioxidant activity of the extracts (Table 2) was highly variable, ranging from approximately 7% in peach flesh to 88% in tomato peels. With the exception of sweet potatoes, the antioxidant activity in the fruit and vegetable peels was in all cases higher than that in the flesh, despite the fact that the carotenoid concentration would often follow the opposite trend. This is likely to be associated with the higher amount of phenolic compounds in the peels, as they have been shown to contain these molecules in higher amounts than the flesh [7,44,45]. This is due to their role of acting as physical and chemical barriers to deterioration caused by mechanical injuries or fungal infections. The use of ethanol as a co-solvent in SFE increased the polarity of CO_2_ and this has possibly led to the extraction of phenolic compounds, as they are also known to exert high levels of antioxidant activity, sometimes even higher than that of carotenoids [7].

An important finding in this work is the fact that the mathematical model developed to identify the optimum conditions for maximum extraction of carotenoids from carrot peels was applicable for a variety of fruit and vegetable matrices, including a mix thereof. The ability of the model to assess the recovery of total carotenoids from each individual matrix as well as in the mix sample was presented in Table 2. The model took into account three parameters to predict the total carotenoid recovery, namely temperature, pressure and co-solvent (ethanol) concentration. In order to better understand the probable reasons behind this, data from optimisation studies for carotenoid extraction by SFE from different fruit and vegetables carried out in the last 15 years were compiled and are presented in Table 4. In all these studies, a number of parameters were investigated (e.g., temperature, pressure, solvent flow rate, co-solvent concentration, extraction time, mass loading, etc.), and various responses measured (yields or recoveries, total carotenoid concentration), whereas the statistical methods employed to develop models varied as well. A direct comparison of the results obtained in this work to these in the literature is challenging, as their presentation can vary considerably. Some authors report the carotenoid data as concentration—the carotenoid mass in the extract per extract mass—or as the carotenoid mass in the extract per raw (dried or wet) sample mass. However, a more efficient way to present the results would be as % recovery (% of total of carotenoids extracted from the total carotenoid mass originally in the raw samples), as this would enable a direct comparison between different samples, processes and extraction protocols. Unfortunately, not all authors report such data.

Despite the aforementioned limitations, a deeper assessment of the literature data in relation to that generated in this work still allows for interesting conclusions. For instance, the most common co-solvent employed is ethanol, which usually ranges from 5 to 15% *v/v*. The preference for this particular entrainer is due to its low price and toxicity but also to its ability in increasing the polarity of CO_2_, compared to other polar solvents, such as methanol or acetone. In addition to these solvents, some authors used vegetable oils as co-solvents (e.g., canola, olive, hazelnut, sunflower oil), due to their positive effect on the solubility of carotenoids, since these molecules are highly lipophilic. The apparent solubility of lycopene was calculated for a solvent mixture of consisting of supercritical CO_2_ and canola oil in an earlier work [12], and it was concluded that the solubility of carotenoids when oil was used as a co-solvent was higher than when ethanol was used or only pure supercritical CO_2_ was employed. Another work also reported very good yields when using canola oil as a co-solvent for extracting carotenoids from carrots [10], due to penetration of the oil into the cell structure of dried carrots, which in turn caused its swelling and enabled the diffusion of supercritical CO_2_ through the matrix. However, these studies did not report the % recovery of total carotenoids, and it is therefore difficult to compare these to the results of this study. The ethanol concentration used here (15.5% *v/v*), which was predicted by the model as the optimum value for SFE of carrot peels, can be classified at the upper end of the concentration range usually used for SFE and is likely to have improved the carotenoid recoveries compared to lower or null ethanol concentrations. That was probably not only owing to a significant increase in polarity but primarily, to facilitating the dissolution of larger molecules, such as carotenoids, in the supercritical solvent. This is also most likely one of the reasons contributing to the high carotenoid recoveries obtained for all the different fruit and vegetable matrices tested in the present work as well.

Pressures usually used in SFE range from 127 to 507 bar, while temperatures range from 40 to 90 °C. The use of high temperatures is limited due the thermolability of carotenoids, as high temperatures are known to cause their degradation and isomerisation [57]. The parameters of pressure and temperature together dictate the solvation power of CO_2_ and are usually considered to significantly influence the extraction process. From the data in Table 4, it can be observed that mid- to high temperatures (between 55 and 70 °C) and high pressures (between 300 and 450 bar) usually result in higher carotenoid recoveries. The general consensus is that the pressure is a more influential parameter than temperature, and pressures above 250 bar have been shown to positively influence carotenoid extraction. This can be explained by the fact that higher pressures result in a higher solubility of carotenoids in the solvent due to increased solvent density. The settings used in this work, i.e., pressure 350 bar and temperature 59 °C, which were predicted by the model as the optimal values for carotenoid extraction from carrot peels, were within these boundaries, which again reinforce the applicability of the model to a wide range of fruit and vegetable matrices. Some studies found that when the pressure was increased above 350 bar, the recoveries of lycopene, α- and β-carotene decreased compared to when lower pressures were employed [48,58,59]. This may be due to the increased polarity of the supercritical fluid solvent mixture (CO_2_ and polar solvents) caused by a significant density increase, which decreased the affinity of the solvent for non-polar molecules, consequently diminishing its selectivity [57].

The extraction time (t_E_) and solvent flow rate (Q) have also been reported to influence the SFE process for fruit and vegetable matrices. The values reported in the literature vary significantly (Q from 0.85 to 3175 g/min and t_E_ from 30 to 720 min). The general trend is that longer extraction times can lead to decreased carotenoid recovery, possibly due to degradation reactions [60,61]. Therefore, an increased solvent flow rate (e.g., higher than 10 g/min) should be used to decrease the extraction time [10]. In this work, a flow rate of 15 g/min was used, enabling the SFE protocol to be completed within 30 min, which is advantageous from the perspective of an industrial process, since shorter processing times imply lower energy consumption and higher productivities. As these two parameters seem to be influenced by the type of the food matrix, optimisation studies are required to pinpoint these parameters for a particular type of matrices.

The influence of other process variables on the recovery of carotenoids from fruit and vegetable matrices is more difficult to predict. For instance, the amount of sample loading in the supercritical fluid extractor varies greatly in the literature (0.5 to 720 g) due to a number of reasons. The most apparent is equipment dimensions, since extraction vessels are available in a range of different volumes, limiting the sample mass to a certain amount. Additionally, some of the works cited were performed in lab-scale equipment, where factors such as particle aggregation and bed geometry are not so important and therefore negligible, whereas others were performed using pilot-scale extraction vessels, where these factors have a considerable influence on the final results.

Finally, in terms of total carotenoid recovery (TCR), it can be seen that the recovery values obtained in this work for a number of fruit and vegetable matrices are higher than those reported in the literature. The extraction of pumpkin flesh by SFE was reported to result in a TCR of 74% at 70 °C and 350 bar [50] and 76% at 50 °C and 250 bar [9], while in the current study, this was 89%. The application of an intermediate temperature (59 °C) compared to the temperature used in these published works seems to have increased the yields considerably. For tomatoes, the TCR values reported in the literature were of 72% for flesh [55] and 33% peels [17], whereas in this work, the TCR for tomato flesh was 92% and for peels 91%. One possible reason for the low recovery observed for the tomato peels in the case of the published work could be the very high flow rate employed (the highest used amongst all published works), which most likely resulted in a very low residence time for the solvent within the extraction vessel and consequently, in low mass transfer rates.

In conclusion, the high carotenoid recoveries indicate that the developed model can be used as a general model for the extraction of carotenoids from various fruit and vegetable matrices by SFE. Moreover, the SFE process was able to extract carotenoids from a mixed sample of fruit and vegetable matrices, with high recovery levels (74% to 99% *w/w*). It is worth pointing out that in order to attest the full feasibility of the described process on a real industrial setting, as hinted with the mix of matrices tested, scale-up studies should be carried out. Factors that can often be neglected when working under lab-scale conditions can come into effect when working with much larger amounts of sample, as is the case in a vegetable processing company. However, the findings here demonstrated that SFE can be used as a viable alternative to conventional solvent-based extraction techniques and potentially as a viable method for adding value to fruit and vegetable waste streams by generating carotenoid-rich extracts from zero or negative cost materials.

## 3. Materials and Methods

### 3.1. Sample Preparation

Fifteen matrices of carotenoid-rich fruits and vegetables were tested, all purchased from a local supermarket chain in Reading (UK). These included the flesh and peels of sweet potato (*Ipomoea batatas, var. Beauregard*), red tomato (*Lycopersicon esculentum Mill., var. Sungold*), apricot (*Prunus armeniaca, var. Moorpark*), pumpkin (*Cucurbita pepo, var. Cinderella*) and peach (*Prunus persica, var. freestone*); green, yellow and red bell peppers (*Capsicum annuum*) and their waste residues (seeds and stems), as well as a mix of all these different matrices (using the same amount of each vegetable) to simulate an industrial scenario of a fruit and vegetable processing establishment.

All vegetables were washed and peeled manually. The samples were then frozen at −20 °C for 36–48 h, freeze dried (VirTis SP Scientific, Ipswich, UK) for 72 h, milled with a home grinder for 2 min and sieved to cut off particles greater than 750 μm in diameter. The samples were then stored in containers away from light and kept at −20 °C until further analysis.

### 3.2. Supercritical Fluid Extraction

For each run, 5.0 g of freeze-dried samples were placed in a supercritical fluid extractor (SciMed, Stockport, UK). A total of 95.0 g of inert glass beads (Sigma-Aldrich, Dorset, UK) were added to fill the vessel volume in order to avoid dispersion effects and the samples submitted to a CO_2_ flow rate of 15 g/min and the dynamic extraction time was fixed at 30 min. These operating conditions were previously optimised for carrot peels via a Central Composite Design of Experiments [23] and included: temperature of 59.0 °C, pressure of 350 bar and 15.5% (*v/v*) of ethanol as co-solvent. Runs were performed in duplicates and the results are presented as the average value for all measurements. The extracts were collected dissolved in ethanol and stored at −18 °C in dark glass containers until further analysis.

### 3.3. Moisture Content

The moisture content in the samples was measured by a halogen moisture analyser (Mettler Toledo, UK). The apparatus was equipped with an oven operating at 105 °C and a precision scale, which determined the water content by gravimetry from the sorption isotherms plotted by the equipment.

### 3.4. Carotenoid Extraction and Analysis

The carotenoid content of the initial fruit and vegetable samples were analysed according to the protocol described by Biehler et al. [62] for non-saponified samples. Briefly, 1.0–2.0 g of freeze-dried samples, both of flesh and peel, were weighed and added to 6 mL of methanol. After vigorous mixing, samples were centrifuged for 5 min at 2500× *g* and the supernatant was separated; a new extraction was performed twice with 8 mL of a mixture of hexane and acetone (1:1). Subsequently, the organic solvent fractions were combined, 25 mL of saturated NaCl were added, and the mixture was shaken in a separator funnel. After phase separation, the lower, water-phase was re-extracted with 8 mL of hexane and the resulting supernatant was combined with the first. The combined fractions were evaporated under nitrogen stream and re-dissolved in methanol prior to High Pressure Liquid Chromatography (HPLC) analysis, as described in the next paragraph. The SFE extracts, in turn, obtained dissolved in the ethanol used as co-solvent, were directly filtered and submitted to the HPLC analysis.

The HPLC analysis was performed with an Agilent Infinity 1260 series HPLC system coupled with a 1260 DAD detector (Agilent Technologies, UK). An YMC-C30 silica-based reversed-phase column (250 × 4.6 mm) was used in the separation of carotenoids with a gradient method consisting of (A) methanol/MTBE/water (82:16:2) and (B) methanol/MTBE/water (23:75:2) as mobile phase. The gradient started at 100% of A. Solvent B was then increased to 50% (0–45 min) and further increased to 100% (46–55 min), with this condition being held for 5 min, totalling 60 min per run. The injection volume was 100 μL and the flow rate was kept constant at 1.0 mL/min. In the case of the carotenoid extracts, the aliquots were directly filtered and injected in the HPLC. For carotenoid identification and quantification, previously-built calibration curves of external commercial standards (α-carotene, β-carotene, lutein and lycopene; Sigma-Aldrich) were used. All detected peaks were analysed at 450 nm.

### 3.5. Total Lignin

The total lignin content was determined according to the NREL protocol [63]. Approximately 300 mg of sample were submitted to acid hydrolysis with 3 mL of H_2_SO_4_ (72%, *v/v*) followed by incubation at 30 °C for 1 h. The liquid phase was then diluted to 3% H_2_SO_4_ (*v/v*) and autoclaved at 121 °C for 30 min. Samples were then filtered then vacuum filtered through pre-weighed filter crucibles and the filtrate was measured for acid soluble lignin spectrophotometrically (Thermoelection Corp., UK) at 240 nm. The washed residue was dried at 105 °C for 18 h. Subsequently, the dried samples were placed in a furnace (500 °C; 5 h) and the ash was weighed and classified as acid insoluble lignin. Total lignin was calculated as the sum of acid soluble and acid insoluble lignin.

### 3.6. Antioxidant Activity

The DPPH (2,2-diphenyl-1-picrylhydrazyl) method was used to measure the antioxidant activity with some modifications [64]. Having found very similar results between methanol and buffered methanol, the DPPH solution was prepared by mixing methanol with DPPH reagent (Sigma Aldrich, Sigma-Aldrich, Dorset, UK) at a molarity of 100 μM to ensure maximum absorbances of around 1.0. For the analysis, 200 μL of the extracts (in triplicate) were mixed with 2 mL of the DPPH solution. The mixture was incubated for 30 min in the dark and the absorbance was measured at 517 nm using a spectrophotometer (Thermoelection Corp., Nottingham, UK). The antioxidant activity values were expressed as the percentage of absorbance change, by comparing the absorbance of individual samples against that of the control (200 μL of methanol +2 mL of DPPH reagent).

## Figures and Tables

**Table 1:** Moisture and carotenoid composition of samples after conventional solvent extraction (experimental) and chemical macro-composition (literature data).

Sample	Moisture (% *w/w*)	Carotenoid Concentration (μg/g Dry Weight Basis) Experimental Data					Compositional Data (% g/g, Dry Weight Basis) Literature Data			
		BCar	ACar	Lut	Lyc	TCC	Carb	Protein	Lipid	Reference
SPF	5.3	383.7 ± 21.0	-	46.9 ± 6.7	-	430.6 ± 27.7	80.5	12.2	4.1	[35]
SPP	4.9	144.2 ± 9.4	-	20.9 ± 5.2	-	165.1 ± 14.6	85.5	5.1	4.4	[36]
TMF	8.1	91.0 ± 8.4	-	25.4 ± 5.1	113.2 ± 9.3	229.6 ± 22.8	45.3	25.9	14.5	[37]
TMP	6.2	154.4 ± 9.7	-	16.6 ± 4.0	82.5 ± 7.3	253.5 ± 21.0	93.2	1.9	1.6	[38]
APF	7.2	132.7 ± 9.1	-	-	-	132.7 ± 9.1	89.9	2.4	2.9	[39]
APP	6.8	212.4 ± 14.0	72.7 ± 10.3	-	-	285.1 ± 24.3	-	-	-	-
PKF	5.9	239.6 ± 13.6	-	-	145.7 ± 12.0	383.3 ± 23.6	88.3	6.1	1.6	[40]
PKP	5.3	49.3 ± 9.6	-	-	92.7 ± 8.4	142.0 ± 18.0	86.3	12.2	5.3	[40]
PCF	6.6	20.4 ± 5.2	-	-	-	20.4 ± 5.2	-	-	-	-
PCP	7.0	47.2 ± 6.3	-	12.3 ± 3.9	-	59.5 ± 10.2	-	-	-	-
GPF	7.9	56.7 ± 6.6	-	262.3 ± 15.6	-	319.0 ± 22.2	55.5	10.3	5.1	[31]
YPF	7.7	31.9 ± 4.9	-	205.2 ± 11.4	-	237.1 ± 16.3	55.8	12.3	6.0	[31]
RPF	7.9	-	-	66.7 ± 7.1	-	66.7 ± 7.1	55.4	13.5	5.1	[31]
XPW	5.2	18.3 ± 4.2	-	90.9 ± 8.4	-	109.2 ± 12.6	61.7	29.1	4.5	[41]
MIX	6.1	133.9 ± 11.3	-	46.2 ± 7.0	23.3 ± 7.6	203.4 ± 25.9	-	-	-	This work

SPF = sweet potato flesh; SPP = sweet potato peels; TMF = tomato flesh; TMP = tomato peels; APF = apricot flesh; APP = apricot peels; PKF = pumpkin flesh; PKP = pumpkin peels; PCF = peach flesh; PCP = peach peels; GPF = green pepper flesh; YPF = yellow pepper flesh; RPF = red pepper flesh; XPW = pepper wastes; MIX = mixed samples. BCar = β-carotene; ACar = α-Carotene; Lut = lutein; Lyc = lycopene.

**Table 2:** Recovery (%, *w/w* d.b.) of individual and total carotenoids from different fruit and vegetable matrices in the supercritical fluid extraction (SFE) extract.

Sample	BCar	ACar	Lut	Lyc	TCR (%)	AA (%)
SPF	99.4 *±* 2.6	-	79.9 *±* 3.7	-	97.4	36.6 *±* 2.0
SPP	99.8 *±* 2.9	-	68.2 *±* 3.8	-	95.9	20.7 *±* 1.8
TMF	99.0 *±* 2.8	-	36.2 *±* 6.2	98.5 *±* 2.1	91.8	30.4 *±* 1.7
TMP	96.9 *±* 1.7	-	29.5 *±* 5.8	92.5 *±* 2.2	91.0	87.9 *±* 1.6
APF	99.0 *±* 2.6	-	-	-	99.0	39.2 *±* 2.2
APP	98.7 *±* 3.1	97.9 *±* 2.7	-	-	98.5	51.9 *±* 2.2
PKF	92.4 *±* 3.5	-	-	87.4 *±* 3.1	89.1	42.3 *±* 1.1
PKP	88.2 *±* 3.6	-	-	83.2 *±* 2.5	84.9	77.1 *±* 1.0
PCF	99.8 *±* 3.1	-	-	-	99.8	7.0 *±* 5.1
PCP	99.2 *±* 2.6	-	75.3 *±* 3.9	-	94.2	34.1 *±* 3.1
GPF	98.6 *±* 2.1	-	99.8 *±* 1.1	-	99.6	17.5 *±* 4.6
YPF	99.8 *±* 2.5	-	99.6 *±* 1.8	-	99.6	49.7 *±* 0.9
RPF	-	-	98.1 *±* 2.2	-	98.1	46.5 *±* 2.7
XPW	96.7 *±* 1.8	-	94.5 *±* 2.1	-	94.9	19.0 *±* 3.4
MIX	98.9 *±* 2.4	96.3 *±* 3.9	73.9 *±* 3.0	91.0 *±* 2.5	92.5	57.7 *±* 2.5

Process conditions: T = 59 °C, Pressure = 350 bar, EtOH = 15.5%, CO_2_ flow rate = 15 g/min, run time: 30 min; TCR: total carotenoid recovery, AA: antioxidant activity. BCar = β-carotene; ACar = α-Carotene; Lut = lutein; Lyc = lycopene; SPF = sweet potato flesh; SPP = sweet potato peels; TMF = tomato flesh; TMP = tomato peels; APF = apricot flesh; APP = apricot peels; PKF = pumpkin flesh; PKP = pumpkin peels; PFC = peach flesh; PCP = peach peels; GPF = green pepper flesh; YPF = yellow pepper flesh; RPF = red pepper flesh; XPW = pepper wastes; MIX = sample mix.

**Table 3:** Total lignin, carotenoid recovery (TCR) and moisture content of some of the vegetable matrices assessed in this work.

	Total Lignin (%)	TCR (%)	Moisture (%)
SPF	3.13	97.4	5.3
RPF	10.1	98.1	7.9
YPF	10.9	99.6	7.7
SPP	11.8	95.9	4.9
MIX	14.9	92.5	6.1
PKP	16.3	84.9	5.3

SPF = sweet potato flesh; RPF = red pepper flesh; YPF = yellow pepper flesh; SPP = sweet potato peels; MIX = sample mix; PKP = pumpkin peels.

**Table 4:** Literature data on optimal process parameters, carotenoids recoveries and optimisation methods for the extraction of carotenoids from various fruit and vegetable matrices by SFE.

Food Matrix	Target Compd.	Mass Load (g)	Optimised Extraction Parameters					Carotenoid Recovery (%, *w/w*)	Carotenoid Content (mg/g ext)	Statistical Models/Var. of Influence	Refer.
			CoSol (%)	T (°C)	P (bar)	Q CO_2_ (g/min)	t_E_ (min)				
Apricot bagasse	BCar	2.0	-	60	304	0.85	90	-	-	ANOVA/Temp, Press, PSize, Temp	[21]
Apricot pomace	BCar	1.0	27.4% EtOH	69	311	1.6	90	-	0.098	Central Composite/Temp, Press, CoSol	[22]
Carrot	TC	2.0	5% Canola oil	70	551	1847	240	-	1.91	ANOVA/Temp, Press	[10]
Carrot Peels	TC	50	15.5% EtOH	59	350	15	30	96.2	-	Central Composite/Temp, Press, CoSol	[23]
Citrus press cake	TC, BCrip	1.0	5% to 20% EtOH	60	310	2.1	20	38.0 (TC)/73.0 (BCrip)	0.550	Central Composite/Press, CoSol	[46]
Citrus waste	TC	100	-	45	252	27	120	-	1.98	Box-Behnken/Temp, Press, Ratio	[47]
Paprika	TC	720	-	80	500	415	-	85.0	1.15	Press, Moist	[48]
Pitanga	TC	5.6	-	60	250	4.1	120	55.0	5.47	-	[49]
Pumpkin	TC	0.4	0% to 10% EtOH	70	350	1.24	-	74.0	0.110	Central Composite/Temp, Press, CoSol	[50]
Pumpkin	ACar, BCar, LUT	2.0	10% EtOH +10% Olive Oil	50	250	1.25	-	76.0	0.472	One-way ANOVA/CoSol, Temp	[9]
Pumpkin flesh, seeds	BCar	100	6.0 g EtOH	48	300	212	60	-	0.205	Box-Behnken	[51]
Red bell pepper (flesh)	CAP	5.0	-	50	400	2.5	210	15	0.100	-	[52]
Red bell pepper (waste)	BCar	30.0	-	60	240	25.8	120	68.1	-	Temp, Press, PS	[53]
Spinach	BCar, LUT	500	-	40	350	60	360	-	17.2	-	[54]
Tomato	LYC	24	Enzyme-aided	86	500	3.44	270	38.0	-	Enzyme activity	[11]
Tomato	LYC	-	10% Hazelnut oil	65	425	230	480	72.5	11.6	-	[55]
Tomato	BCar, LYC	10	5% Canola oil	40	400	478	720	-	6.60	-	[12]
Tomato juice	LYC	15	-	80	350	1.7	180	77.0	-	One-way ANOVA/Temp, press, CoSol	[56]
Tomato peel	LYC	1.2	14.0% EtOH	62	450	3175	30	33.0	-	Central Composite Rotatable/Temp, Press	[17]
Watermelon	LYC	0.5	15.0% EtOH	70	207	1.0	35	-	38.0 (w.b.)	Temp	[14]

CoSol = Co-solvent concentration; EtOH = Ethanol; MeOH = Methanol; QCO_2_ = Solvent flow rate; tE = Extraction time; TC = Total carotenoids; ACar = α-carotene; BCar = β-carotene; LYC = lycopene; LUT = Lutein; CAP = Capsanthin; BCrip = β-cryptoxanthin; Var. of inf. = variables of influence; CoSol = Co-solvent; Temp = Temperature; Press = Pressure; Moist = Moisture; PSize = Particle Size; w.b. = wet basis.
